# Long-Term Ecological Impacts of Norway Spruce Plantations on Biodiversity and Microhabitat Conditions

**DOI:** 10.1007/s10021-026-01048-0

**Published:** 2026-03-30

**Authors:** Simone Balestra, Vanessa Manuzi, Erica Ceresa, Pietro Gatti, Reetta Aleksandra Pirttilahti, Jiri Hodecek, Thierry Adatte, Brahimsamba Bomou, Paolo Gabrieli, Stephanie Grand, Gianalberto Losapio

**Affiliations:** 1 Department of Biosciences, University of Milan, Via Celoria 26, 20133 Milan, Italy; 2 Institute of Earth Surface Dynamics, Faculty of Geosciences and Environment, University of Lausanne, UNIL Mouline, 1015 Lausanne, Switzerland; 3 Institute of Earth Sciences, Faculty of Geosciences and Environment, University of Lausanne, UNIL Mouline, 1015 Lausanne, Switzerland

**Keywords:** Arthropods, Biodiversity, Ecological indicator values, Landscape management, Monoculture, Nutrient cycling, Plant ecology, Reforestation, Soil acidification, Tree planting

## Abstract

Reforestation efforts have sought to counteract deforestation and to provide a nature-based solution against climate change. However, they often involve monoculture plantations of non-native species, which may have unintended ecological consequences. Yet, the long-term ecological impacts of planting trees have been poorly estimated. Leveraging historical reforestation conducted in northern Italy during the 1930s by the fascist regime, we assessed the long-term impacts of Norway spruce (*Picea abies*) monoculture plantations on biodiversity of plants and soil fauna along with soil properties. We found that plant diversity in tree plantations was 50.3% lower than in native forests and 74.5% lower than in grasslands. Ecological indicator values for temperature and light were reduced in tree plantations. Additionally, functional evenness was reduced by 30% in spruce plantations, suggesting lower ecological stability. In tree plantations, organic carbon content was 25% higher due to litter deposition and slower decomposition rates. Soil fauna diversity was marginally less affected, suggesting a faster recovery over the last one-hundred years of arthropods as compared to plants. These findings highlight the need for monitoring reforestation interventions, suggesting strategies that incorporate diverse tree species rather than planting tree monoculture to support functionally and resilient ecosystems.

## Introduction

Forests play a crucial role in supporting global biodiversity by providing habitats for 50–80% of all terrestrial organisms (Leuschner and Homeier 2022). Furthermore, forests improve ecosystem functions by contributing to the regulation of the global climate through evapotranspiration and carbon cycling (Feng and others 2024). Yet, deforestation and biodiversity loss continue to threaten the integrity and stability of forest ecosystems (IPBES 2019). Over the past few years, reforestation initiatives have been increasingly implemented to mitigate forest losses and with the aim of mitigating the effects of climate change (Food and Agriculture Organization of the United Nations and United Nations Environment Programme 2020). While reforesting and restoring degraded, deforested land is necessary for enhancing biodiversity recovery and mitigating climate change (Food and Agriculture Organization of the United Nations 2020), half of the area pledged for forest restoration comprises monoculture plantations characterized by non-native tree species (Lewis and others 2019). Such plantations can have negative consequences for biodiversity, soil health, and ecological resilience (Messier and others 2022) as timber production is maximized over biodiversity maintenance (Lewis and others 2019). However, the long-term ecological effects of reforestation on biodiversity remain poorly quantified.

Two key issues of reforestation programs are represented by (1) the poor species diversity and (2) the type and origin of trees used, which involves non-native species. Native forests are rich and complex ecosystems that provide a wide range of services and nature’s contributions to people (Cavanagh and Benjaminsen 2014). Through reforestation, they are often replaced by tree monocultures of non-native or extra-range species. Despite these monocultures might develop into native forests after several decades (Hilmers and others 2018), such as Scots pine (*Pinus sylvestris*) on sandy soils (Diers and others 2024), they often display high mortality rates due to stresses induced by climatic conditions and pathogens (Dimitrova and others 2022). On one hand, funding bodies tend to prefer material ecosystem services, including timber provision, over regulating services such as water retention. On the other hand, reforestation plans are often promoted to compensate for fossil fuel emissions by companies with large responsibilities for CO_2_ emissions, such as airlines. Hence, a few non-native and fast-growing trees, like *Pinus* and *Eucalyptus*, are vastly employed in plantation arrays worldwide (Uribe and others 2021; Liang and Zong-Qiang 2009). Recent studies (Feng and others 2022; Grossman and others 2018; Hua and others 2022) reveal the benefits of multi-species plantations in terms of productivity, stability, and biodiversity maintenance (Ampoorter and others 2020). Valuing plant diversity in reforestation programs might require a greater initial effort, but it would provide a much better outcome in terms of ecosystem health and services in the long term (Liang and others 2025). Monitoring the long-term consequences of reforestation for biodiversity is, therefore, key to ensuring the wider sustainability of forest restoration actions (Richter and others 2024).

Reforestation programs in Italy have been implemented since the beginning of the twentieth century as part of hydro-geological mitigation and recovery plans. The main drivers were associated with the socio-economic situation of that time, characterized by the need for energy resources that could be obtained from timber (Agnoletti and others 2013). Political drivers, including the power of the timber industry and the repression of farmers, played a key role in the plantation programs established by the fascist regime (Armiero and others 2022). These plantations were mostly carried out at the expense of agricultural land, with trees planted in pastures and meadows. In the Italian Prealps, land-use legacies have generated a fine-scale mosaic of forested and open habitats, including long-established pastures maintained through traditional agro-pastoral practices. Although anthropogenic in origin, these semi-natural grasslands have persisted for centuries and host remarkably high levels of biodiversity, often representing regional hotspots for plants and invertebrates. Indeed, they are widely recognized as reference ecosystems for biodiversity conservation and habitat monitoring in mountain landscapes (see Habitats Directive 92/43/EEC).

The choice of planting species was based on generalist and fast-growing species. In the mountains of northern Italy, the species adopted in tree plantations was the Norway spruce (*Picea abies*) due to its fast growth rate and timber quality. These traits promoted its widespread planting in many areas outside of his natural distribution range and habitats (Caudullo and others 2016). The main consequence of these Norway spruce plantations is the creation of a new habitat dominated by a single out-of-range species. Norway spruce is a non-deciduous tree that maintains a constant low light intensity in the understory throughout the year (Manuzi and others 2025). Furthermore, it has an impact on soil conditions by altering the humus layer and litter quality (Ranger and Nys 1994; Augusto and others 2002). These impacts could influence soil chemistry and, in turn, modify the communities of plants and soil organisms. Its effects on soil acidification could be exacerbated in areas with alkaline, carbonate substrates, contributing to the exclusion of native species adapted to more basic soil conditions that cannot withstand soil acidity. However, the impacts of Norway spruce plantations on biodiversity and soil conditions remain poorly monitored in the long-term. It is reasonable to hypothesize that spruce plantations impact biodiversity via two main pathways: directly, by removing native forests in favor of tree monocultures; and indirectly, by changing microhabitat conditions such as soil acidity and nutrient availability.

### Research aim and Hypothesis

The aim of this research is to assess how tree monoculture plantations influence biodiversity in the long-term (*c*. 100 years). Our first hypothesis is that the introduction of a new artificial forest, composed only of Norway spruce, has a negative impact on the biodiversity of plants and soil arthropods. We expect a shift in the species composition of plant and arthropod communities, characterized by the exclusion of native temperate species, but not by replacement with boreal species (Manuzi and others 2025). We also expect a negative impact of Norway spruce monoculture on plant functional diversity and the ecological conditions associated with light availability and temperature. Finally, we hypothesize that Norway spruce litter will drive an accumulation of organic matter and an increase in soil acidification (Perkovi´c and others 2007; Vesterdal and others 2008; Binkley and Valentine 1991), which mediates changes in plant and animal communities. To address these hypotheses, we investigated biodiversity changes by examining shifts in the richness and composition of plant and arthropod communities and by linking those changes to microhabitat and soil conditions.

## Methods

### Study Design

We designed the study to ensure a comparison between the spruce monoculture and the semi-natural habitats in the study area, namely stable secondary forests and pasture–meadows. Despite both habitats requiring human management for their maintenance (particularly the meadow), they can be distinguished from the spruce monoculture by several differences. Firstly, the human management of mountain areas in northern Italy has ancient origins, since meadows and managed forests are semi-natural systems whose origins date back to the Neolithic (Agnoletti 2025; Leuschner and Ellenberg 2017). Deciduous forests represent an example of limited human intervention: management can influence the relative abundance of certain species, but it has not altered the overall structure or the dominant species of the plant community (Banti and others 2015). The meadow is even more related to human management, since its maintenance involves the cutting of existing forests and requires periodic management, like grass-mowing and manuring (Gusmeroli 2012). However, this is an agroecosystem that has existed for thousands of years and represents a hotspot of biodiversity in the Italian Prealps and Europe generally (K¨orner and K`eorner 1999).

In contrast, the spruce monoculture is approximately one century old and is not the result of a gradual, long-term interaction between human management and the ecosystem; rather, it is the artificial establishment of a single, profitable species. Therefore, we included these pasture–meadows (hereafter, grasslands) in our study design to capture the full range of habitat conditions resulting from past afforestation efforts, from open, semi-natural grasslands and mixed native (secondary) forests to spruce monoculture plantations. This gradient reflects the dominant vegetation types and management histories in the region and provides a realistic framework to assess the long-term ecological consequences of spruce plantations on biodiversity and soil characteristics.

**Figure 1:**
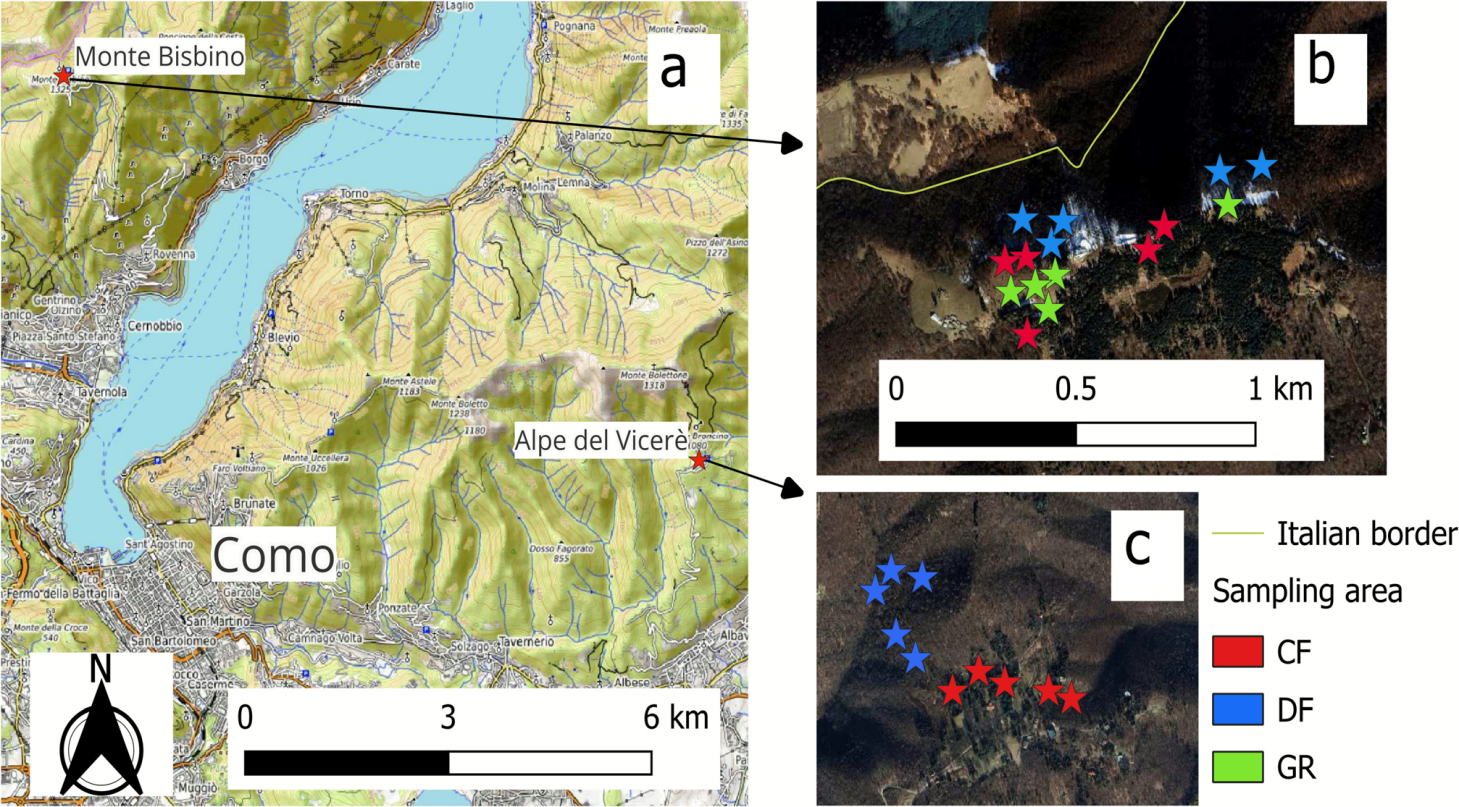
**a**: Location of the study sites. **b**: Satellite image of Monte Bisbino site. **c**: Satellite image of Alpe del Vicer`e site. The satellitary images **b** e **c** represent the fixed plots location. “SM” = Norway spruce monoculture plantations, “DF” = native deciduous forest, and “GR” = grassland (mountain meadow/pasture). Map data: Google, Maxar Technologies.

We examined plant and arthropod diversity as well as soil conditions in a set of permanent quadrants at two sites in the Italian Prealps during the growing season from March to July 2023. The sites (Figure 1) were Mount Bisbino (1300 m asl, Cernobbio, province of Como, Italy) and Alpe del Vicer´e (900 m asl, Albavilla, province of Como, Italy). They are characterized by the same landscape and similar habitat types but are located at different elevations. We adopted the same experimental design in both sites: sampling was conducted regularly over five months to detect species with different phenologies and to monitor changes in plant cover during the growing season. We randomly chose five different plots for each habitat at each site and marked a squared perimeter of 3 × 3 m, 9 m^2^. In total, we sampled 10 plots at Alpe del Vicer`e and 15 plots at Monte Bisbino. A minimum distance of 50 m was maintained between plots and adjacent habitats.

### Study Area

According to SOUISA geological classification (Marazzi 2005), Alpe del Vicer`e is part of the mountain chain “Triangolo Lariano” and Monte Bisbino of the “Tremezzo-Generoso-Gordona” chain, both in the Como prealps (subsections of the Lugano prealps). Both sites rest on the Moltrasio limestone formation (late Jurassic), which is composed of cherty limestones and marls. The bioclimate is temperate, with warm and humid summers and fresh winters (Pesaresi and others 2017); the mean annual temperature is 8.9 °C, mean summer temperature is 18.3° C, and annual precipitation is 1350 mm/year. The semi-natural habitat is characterized by a deciduous forest dominated by *Fagus sylvatica* and *Acer pseudoplatanus* at the Mount Bisbino site, and by *Castanea sativa*, *Tilia cordata* and *Acer sp.* at the Alpe del Vicer`e site. Native forests and grasslands are sparse among reforested areas of Norway spruce. The examined meadows are dominated by *Dactylis glomerata* and *Arrhenatherum elatius*, and have a mixed management that alternates between mowing and grazing.

Mount Bisbino plantations were installed during the 1930s (Bartolini 2016), while Alpe del Vicer´e plantations were installed in 1930–1935 as part of the”Alpine Village” built by the fascist regime, despite there were already present some spruce plantations in neighboring areas (Borghese 1992). In Alpe del Vicer´e site, it was not possible to sample the meadows because they occur at a higher elevation compared to the forests, conditions that would not provide the same bioclimate necessary for a robust comparison.

### Field Surveys

We sampled plant and arthropod diversity across the three habitats of spruce mono-culture plantations, deciduous native forests, and grasslands (Figure 1). We collected plant data taking into account the cover of each species occurring in each plot. Only in spruce monoculture and deciduous forest, we sampled woody plants using a stratified method, which considers three levels of growth related to a specific stage of life history based on stem diameter: seedling (< 1 cm), sapling (1–10 cm), and adult (> 10 cm).

Plots were resurveyed monthly; then, the sampling rounds of plants and arthropods have been aggregated for the statistical analyses. The species accumulation curve for sampling of plants is provided in Appendix A, showing the solidity of our sampling effort and the reliability of inherent results.

We sampled the soil fauna community using a pitfall trap made with thermoplastic polyester (PLA) of 200 ml volume, filled with 50 ml of Propylene Glycol (C_3_H_8_O_2_). The traps were placed in the center of each plot, with the rim at the surface level, to passively trap mainly ground and litter arthropods. We made two cycles of arthropod samplings (*n* = 50 traps), one in April and one in May. The traps were operational for a week. The samples were stored in an alcoholic solution (Et-OH 70%) prior to specimen identification.

The species identification procedure followed different strategies for plants and arthropods. We identified all the 136 surveyed plant species (Appendix B) according to “La Flora d’Italia” (Pignatti and others 2017) and “Flora Alpina” (Aeschimann and Lauber 2004). The arthropods have been observed and sorted in the laboratory under a stereomicroscope (Leica IC90E) using maximum magnification. Adult arthropods were identified by a single identifier based on prior training and experience. When species-level identification was not possible with high confidence, specimens were assigned to the lowest reliable taxonomic rank (typically genus or family) or grouped as morphospecies defined by consistent external characters across the study. We also created a project on the platform *iNaturalist* to take advantage of species identification provided by many experts from Italy and Europe (https://www.inaturalist.org/projects/impact-of-spruce-plantation-on-plant-diversity and https://www.inaturalist.org/projects/impact-of-spruce-plantation-on-arthropod-diversity). More information about the identification and community confirmation on *iNaturalist* can be found in Appendix C. We identified a total of 201 species and morphospecies. The checklist of identified arthropod species is available in Appendix C.

### Soil Analysis

All the sampled plots lie on the same geological substrate of limestones and marls, belonging to the Moltrasio Formation. The soils found in the sampling sites are classified as Cambisols according to WRB criteria (Mantel and others 2023), with a distinction between Humic Cambisols in the deciduous forest, Dystric Cambisols in the coniferous forest and Calcaric Cambisols in the grassland. Since we were interested in plant-soil interactions, we focused our soil analysis on the top two horizons: (1) horizon O, which is located at 2–10 cm of depth and is constituted by plant litter and other organic matter; (2) horizon A, located at 10–30 cm of depth and made by organic mineral complexes.

Two separate soil samples were taken from each plot at 5 cm depth for the horizon “O” and at 20 cm for the horizon “A”. We pooled 100 g of soil sample per horizon from three random spots in the plot. The samples were air-dried for 7 days at room temperature and sieved to remove gravel particles larger than 2 mm, which are not considered fine soil particles. The standard soil analyses were then carried out on sieved soil (Appendix D). For each soil analysis, 20 % of the initial number of samples (*n* = 25) were duplicated in order to assess the relative error and reliability of the measurements (with a total *n* = 30). An error of up to 10 % has been accepted. pH was measured with 6 g of sieved soil in a suspension of deionized water, using a combined electrode pH-meter in accordance with AFNOR standard NF X-31-103, 1998 (Charles and others 2025), with a soil/solution ratio of 1:2.5. As the soil pH did not exceed 6.5 (Figure 6a), no significant carbonates in the < 2 mm fraction are expected (Gozukara and others 2025).

The analysis of organic carbon and nitrogen content (CHN) in soil was performed on 10–20 mg of ground samples. Before detecting soil organic carbon, acid fumigation is carried out to remove soil carbonate (Harris and others 2001). This process involves exposing the soil sample to acid vapors, which react with and remove the carbonate compounds present. This step is crucial to ensure that the measured carbon content reflects the organic carbon content of the soil without interference from inorganic carbonates. Following acid fumigation, the soil sample was prepared for CHN analysis through combustion. To estimate available nutrients in soil the method used for the extraction is the Mehlich methodology (Mehlich 1984). It was done on 1 g (organic samples) to 2 g (mineral samples) of sieved soils. Soil samples undergo ICP (Inductively Coupled Plasma) spectroscopy, that identifies the target elements (Mylavarapu and others 2014). The nutrients obtained here are P, K, Ca, Mg, and Mn.

Finally, to observe the distribution of the inorganic forms of various elements, the X-ray fluorescence (XRF) was carried out. This method allows for determining the proportion of oxides of the following elements: Si, Al, Fe, K, Mg, Ca, Na, Ti, Mn, and P. In this analysis, fused beads have been prepared using dilithium tetraborate (Li_2_B_4_O_7_) and then burned at 1200 °C, to obtain a molten glass pellet. The glass pellet was then subjected to XRF analysis to determine the sample proportions of the various oxides. We chose to consider further only the elements that were expected to be more influenced by the spruce litter, such as carbon, nitrogen, calcium, magnesium and potassium. These elements are good indicators of the change in SOM content (Stevenson 1994). We also considered pH and the amount of exchangeable metal ions of aluminum and iron since they are influenced by soil acidification (Lundstr¨om and others 2000).

### Ecological Indicators and Functional Diversity

Finally, to assess long-term changes in ecological conditions of habitats, we adopted a bioindication approach (Ivanova and Zolotova 2023). To this end, we used Landolt’s Ecological Indicators (EIV) from (Landolt and others 2010) for each and all plant species across habitats. These EIV indicators range from 1 to 5 depending on the species’ ecology. We used the following categories:

- Soil reaction (R): Indicates the level of soil reaction that the plants prefer. It ranges from highly acidic soils (*R* = 1) to ultra-alkaline soils (*R* = 5).
- Temperature (T): Reflects plant’s preference for specific temperature ranges. It ranges from cold-tolerant species that occur in the alpine zone (*T* = 1) to those typical of the lowland zone and hot bioclimate (*T* = 5).
- Moisture (F): Reflects plant’s preference for the moisture content of the soil. It ranges from very dry (*F* = 1) to waterlogged environments (*F* = 5).
- Aeration (D): Refers to the tolerance to soil aeration and compaction. The scale typically ranges from species that tolerate compact or waterlogged soils (*L* = 1) to those that need well-aerated soils (*L* = 5).
- Light (L): Refers to the tolerance and preference of the plant for light exposure. The scale typically ranges from species that thrive in deep shade (*L* = 1) to those that require full sun (*L* = 5).
- Continentality (K): Refers to the response to the amplitude of annual temperature variation. The scale typically ranges from species typical of oceanic climates (*L* = 1) to species typical of continental climates (*L* = 5).
- Continentality (K): Refers to the response to the amplitude of annual temperature variation. The scale typically ranges from species typical of oceanic climates (*L* = 1) to species typical of continental climates (*L* = 5).
- Humus content (H): Reflects plant’s preference for the organic matter content of the soil. It ranges from more mineral soils (*F* = 1) to humus-rich or peat soils (*F* = 5).
- Nutrients (N): Indicates the tolerance or requirement of the plant for soil nutrients. It ranges from oligotrophic (*N* = 1) to eutrophic environments (*N* = 5).

We used EIVs to calculate the community-weighted means of each indicator and functional indices as aggregated measure using the R function”dbFD” from the FD package (Lalibert´e and others 2014; Losapio and others 2025). To this end, we built two matrices as input: one matrix with species in rows and EIV in columns, and a second matrix with plots in rows and plant species in columns. For functional analysis, we considered functional evenness (FEve). FEve measures how evenly the functional traits of a community are distributed, indicating how regularly the species fill the available functional space (Mason and others 2005; Lalibert´e and Legendre 2010; Vill´eger and others 2008). High FEve indicates an efficient and fair use of ecological niches, leading to a more stable ecosystem. On the contrary, a low FEve suggests that the species occupy only some areas of the functional space, with lacks in other traits. This could cause problems in resource use efficiency of the ecosystem.

### Statistical Analysis

Data analysis was conducted in RStudio (version 2023.12.1 + 402.pro1) (Posit Team 2023) using the R packages mixOmics (F and others 2017), lavaan (Rosseel 2012), parameters (Lu¨decke and others 2020), effectsize (Ben-Shachar and others 2020), rfPermute (Archer 2023) and vegan (Oksanen and others 2022).

First, we performed regression analysis to assess how biodiversity and soil change with spruce monoculture as compared to native forests and grasslands. In particular, for plant and arthropod diversity, we tested the response of species richness (two separate models for plants and arthropods) to spruce monoculture (reference level, with native forests and grasslands as conditional levels) using a generalized linear mixed model (GLMM) with a Poisson distribution and site as a random effect. For EIV and FEve, we used linear mixed models (LMM) for each ecological index (Light, Temperature, Moisture, Soil Reaction and Nutrients) including spruce monoculture (reference level, with native forests and grasslands as conditional levels) as fixed effect and site as a random effect. For soil conditions, we used LMM to compare soil parameters among habitats including spruce monoculture (reference level, with native forests and grasslands as conditional levels) and soil horizon (categorical with horizons O and A) as fixed effects, their statistical interaction, and site as a random effect.

Second, we used multivariate statistical analysis (PLSDA and path analysis) to address changes in community composition and their environmental drivers. With PLSDA, we focused on the differences between the monoculture and the semi-natural habitats. PLSDA was performed on a binary matrix with the presence/absence of species as predictors. In this way, we used the species to discriminate among the habitats, also identifying which ones contribute the most to distinguish one habitat from the others.

Third, path analysis was developed starting from a stepwise model with selecion based on AIC criterion and the backward elimination process. We selected two equations with plant diversity and arthropod diversity as dependent variables. We built each equation by including all the variables that were potential predictors: soil parameters, i.e., carbon, nitrogen, calcium, magnesium, aluminum, iron, potassium, and pH; ecological indicators, i.e., CWM of Soil reaction, Temperature, Moisture, Aeration, Light, Continentality, Humus content, and Nutrients; a binary variable of habitat naturalness (1 stands for “spruce monoculture” and 0 for “semi-natural habitat”) and a binary variable of habitat structure (1 for “herbaceous” and 0 for “forest” habitat). For arthropod diversity, we also considered plant diversity as a predictor since plants are a source of food for many arthropod taxa. Stepwise selection removed redundant variables according to the lowest AIC value, which indicates the best fit for our model (Yamashita and others 2007). Finally, the stepwise selection process with backward elimination suggested the following model composed of these two functions (1) and (2):

1 $$ {\mathrm{Plant}}\,{\mathrm{diversity}} \sim C/N\, + \,{\mathrm{Al}}\, + \,{\mathrm{habit}}\,{\mathrm{at}}\,{\mathrm{type}}\, + \,{\mathrm{habit}}\,{\mathrm{at}}\,{\mathrm{origin}} $$

2 $$ {\mathrm{Arthropod}}\,{\mathrm{diversity}} \sim {\mathrm{Plant}}\,{\mathrm{diversity}}\, + \,{\mathrm{Fe}}\, + \,{\mathrm{habit}}\,{\mathrm{at}}\,{\mathrm{type}}\, + \,{\mathrm{habit}}\,{\mathrm{at}}\,{\mathrm{origin}} $$

*C*/*N* is the ratio of the SOC and nitrogen. Al and Fe are the exchangeable elements aluminum and iron, habitat type is a binary variable where 1 stands for grassland and 0 for forest habitat, habitat origin is a binary variable where 1 stands for spruce monoculture and 0 for semi-natural habitat.

The goodness of fit of this model was evaluated considering the set of parameters suggested by Kline (2023) and Lomax (2004).

## Results

### Biodiversity

**Figure 2:**
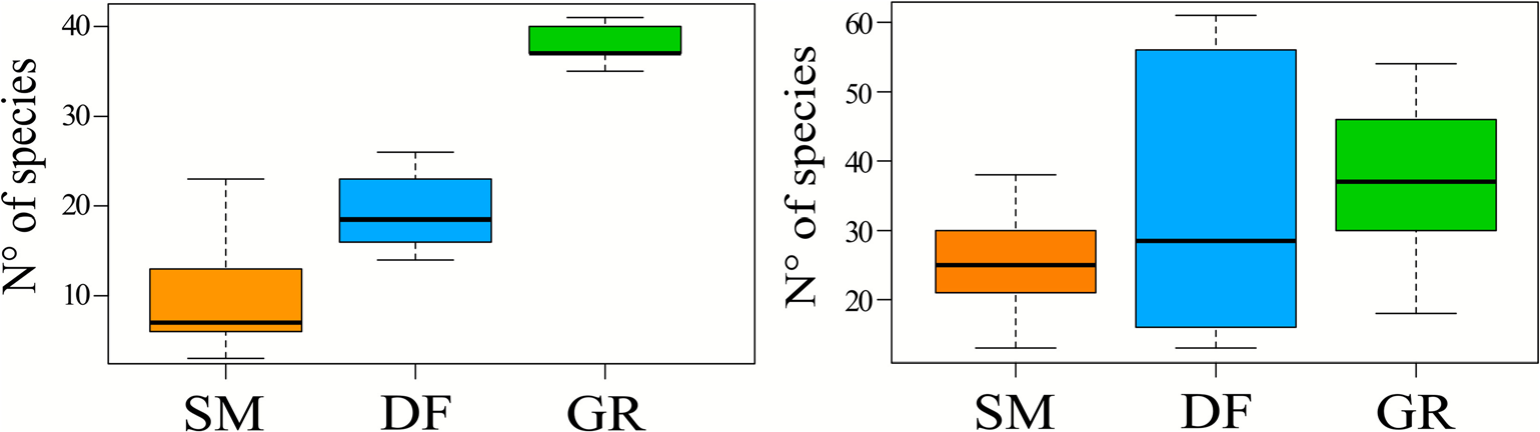
Box plots representing how plant diversity and arthropod diversity change among habitats. On the *x*-axis are reported the three habitats (“SM”: spruce monoculture plantations, “DF”: deciduous forests, “GR”: grasslands), on the *y*-axis biodiversity is measured as number of species. The colorful rectangle represents the IQR (Interquartile Range), that is the central 50% of data distribution. The bold black line represents the median that divides the box in first quartile (lower half) and third quartile (upper half). At the ends of the box there are the whiskers, which indicate the data dispersion outside the IQR without include the outliers.

Plant diversity changed among habitat types (*p *value < 0*.*001), with higher values in grassland and deciduous forests compared to spruce plantations (Figure 2a). Plant diversity is 50.3% lower in spruce plantations than deciduous forests and 74.5% lower than grasslands. The median of species per plot was 7 for monoculture, 18.5 for deciduous forests and 37 for grasslands.

Arthropod diversity was independent of habitats (*p *value = 0*.*24). The median of arthropod taxa per plot was 25 in spruce monoculture plantations, 28.5 in deciduous forests and 37 in grasslands. The IQR of deciduous forests showed a very high variance.

### Partial Least Square Discriminant Analysis

**Figure 3:**
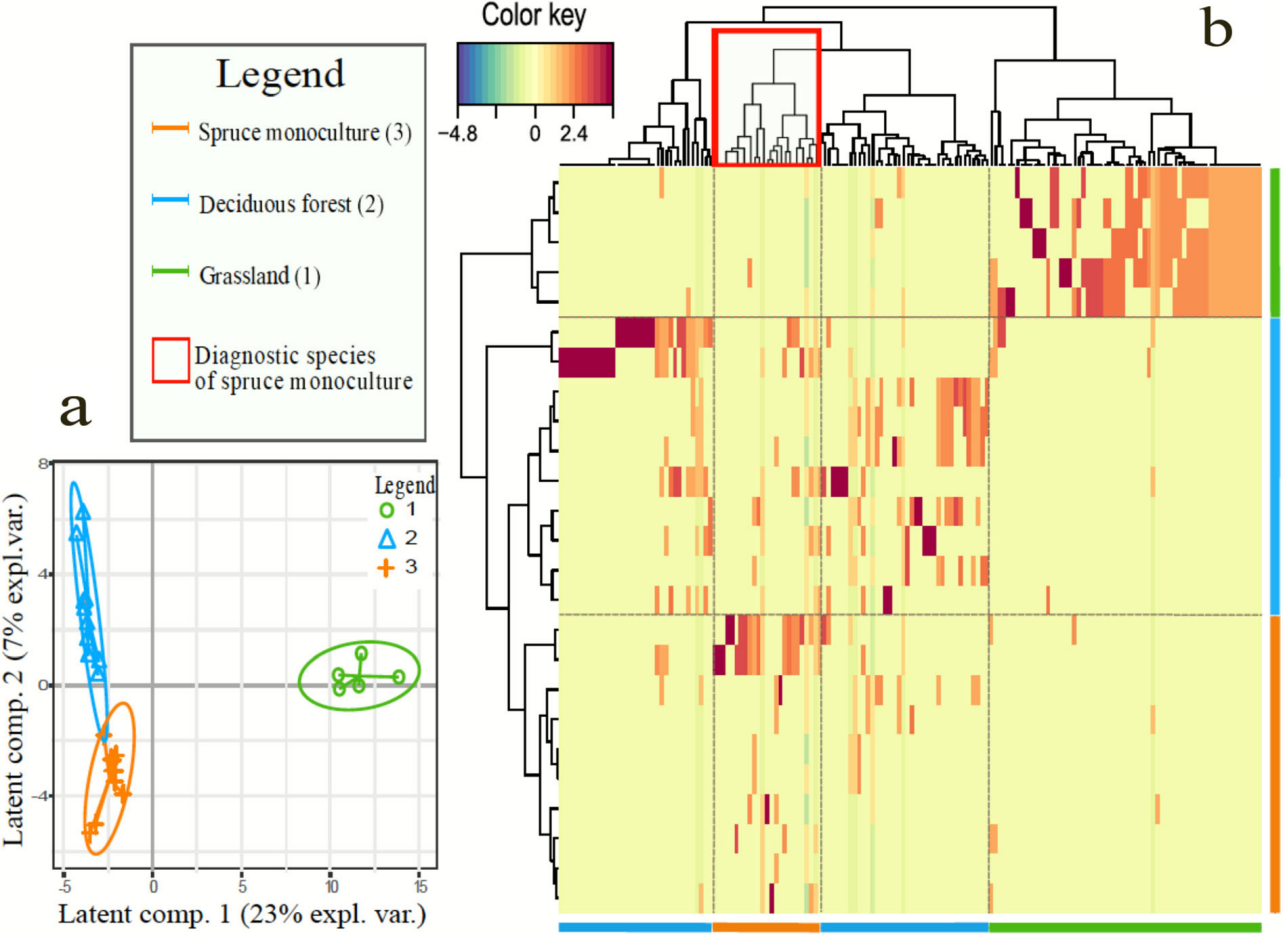
Graphs resulting from the PLSDA analysis of plant communities. **a** Dendrogram representing the differentiation among habitats in terms of community composition. The color key indicates the grade of differentiation among habitats and shows an increase of intensity from blue to red. The colored bars on the sides of the graphic represent the plant community of the three habitats. **b** Biplot with first two components and the percentage of variance explained by the model is reported on the axes.

We produced two graphs: a: dendrogram that highlights the discriminant species among the habitats. b: scatter plot that shows how the plots are distributed in the space of components. In the biplot (Figure 3a), the first and second components show significant differences in species distribution and composition between spruce monoculture and semi-natural habitats (*p *value < 0*.*001 and *p *value = 0*.*005 for first and component, respectively). The first component explained 23% of variance, distinguishing the three habitats from each other. In particular, it shows a strong differentiation among grasslands (green ellipse) and forests (blue and orange ellipses). The second axis explained 7% of variance, differentiating deciduous forests from spruce monoculture plantations.

The dendrograms (Figure 3b) deepen these results, showing reciprocal differentiation of habitats in the first component in the left part of the dendrogram, where the upper branch distinguishes grasslands from the two forest communities. The upper dendrogram shows how the species are clustered depending on their presence/absence in the habitat. This dendrogram distinguishes the pool of species related to grasslands from forest communities. It also provides a more precise picture of how the plant community of spruce monoculture is related to deciduous forests. Plant species of spruce monoculture form a cluster (red rectangle in Figure 3b) included in the same hierarchical level of the clusters related to deciduous forests, while it does not belong to a separate cluster as one would expect in case of habitat differentiation and community turnover. The set of plant species that best differentiate habitats are reported in Table 1.

**Table 1:** Set of Species Identified by the PLSDA Model as Distinguishing Factors of the Plant Communities Across Habitats

Spruce plantations	Deciduous forests	Grasslands
*Primula vulgaris*	*Corylus avellana*	*Arrhenatherum elatius*
*Geranium robertianum*	*Acer pseudoplatanus*	*Ranunculus acris*
*Cardamine bulbifera*	*Veratrum album*	*Thymus sp.*
*Daphne laureola*	*Castanea sativa*	*Achillea millefolium*
*Prunus avium*	*Ranunculus platanifolius*	*Cruciata glabra*
*Rosa sp.*	*Lilium bulbiferum*	*Lathyrus pratensis*
*Geranium nodosum*	*Narcissus poeticus*	*Hippocrepis comosa*
*Brachypodium sylvaticum*	*Lonicera xylosteum*	*Betonica officinalis*
*Urtica dioica*	*Adoxa moschatellina*	*Galium lucidum*
*Larix decidua*	*Sorbus aria*	*Veronica chamaedrys*
*Hieracium sylvaticum*	*Geranium nodosum*	*Potentilla erecta*
*Helleborus viridis* *Ilex aquifolium*	*Asperula taurina* *Senecio nemorensis*	*Hieracium pilosella*

### Ecological Indicator Values (EIVs)

#### EIVs Estimation

**Figure 4:**
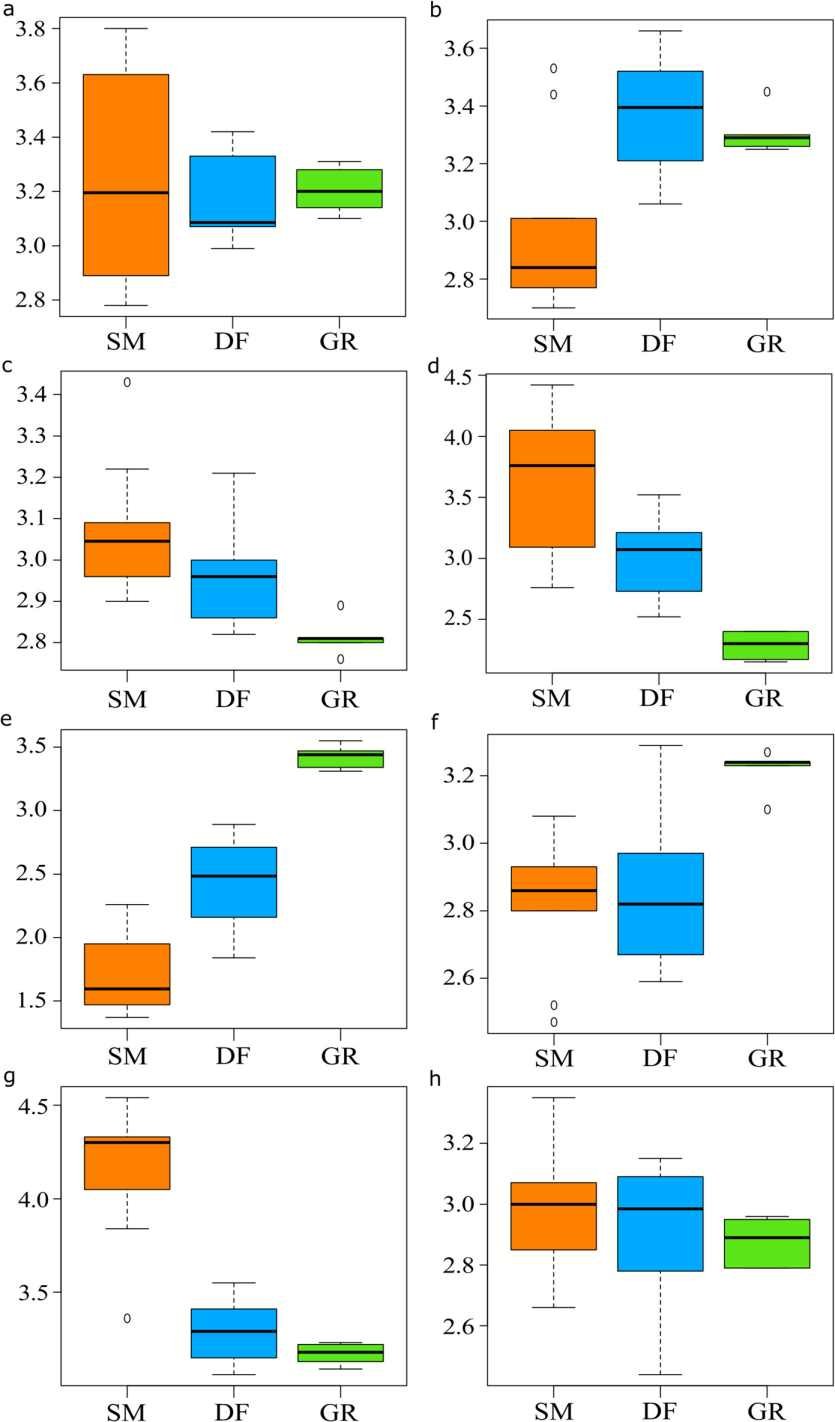
Box plots representing the differences of Landolt’s Ecological Indicator Values (EIVs) among habitats (“SM”: spruce monoculture plantations, “DF”: deciduous forests, “GR”: grasslands). The community means of EIVs are reported on the *y*-axes. Ranges of EIVs are described in detail in Section "Soil Analysis". **a** Ranges from acidic to basic soil. **b** Ranges from low temperature to high temperature. **c** Ranges from dry soil to humid soil. **d** Ranges from compact to aerated soil. **e** Ranges from shadow to light environment. **f** Ranges from oceanic to continental climate. **g** Ranges from mineral to humus-rich soil. **h** Ranges from oligotrophic to eutrophic soil.

Regarding bioclimatic conditions, the EIVs of temperature indicate that deciduous forests and grasslands provide habitats for communities adapted to higher temperatures than those recorded in spruce monoculture plantations (*p *value = 0*.*001; Figure 4b). Species related to spruce appear to be well adapted to colder climatic conditions. The EIVs of light indicate that spruce monoculture provides habitat for communities with lower values than the deciduous forest, which in turn has lower values than grassland (*p* value < 0*.*001; Figure 4e). The species of spruce monoculture plantations are better adapted to lower levels of light than species of deciduous forests and grasslands. The EIVs of continentality indicate that both spruce mono-culture plantations and deciduous forests host a pool of species with a lower value of continentality compared to grasslands (*p* value = 0*.*002; Figure 4f), indicating that plant community of grasslands are well adapted to climates with low precipitation and higher thermal amplitude.

Regarding edaphic conditions, we found that EIVs of soil reaction show no significant differences between spruce monoculture and other habitats (*p  value* = 0*.*86, Figure 4a). The values for spruce monoculture have a high variance, suggesting the presence of a wide range of plant species adapted to grow in different soil pH conditions. The EIVs of soil moisture were similar between spruce monoculture plantations and deciduous forests. These two forest habitats have higher values than grassland (*p *value = 0*.*004; Figure 4c), indicating higher moisture in spruce monoculture plantations and deciduous forests than grasslands. The EIVs of aeration indicate that species occurring in spruce monoculture plantations have deeper roots than deciduous forests and grasslands (*p *value < 0*.*001; Figure 4d). This decreasing trend shows that ecological conditions sustaining aeration are the lowest in grassland. The EIVs of soil humus content suggest that spruce monoculture plantations offer a habitat with higher amount of humus content than deciduous forests and grasslands (*p *value < 0*.*001; Figure 4g). The species of monoculture are therefore expected to be well adapted to soil with slow turnover and high accumulation of organic matter. The EIVs of soil nutrients do not show differences among the three habitats (*p *value = 0*.*41; Figure 4h). The additional information on the model statistics is summarized in Table 2.

**Table 2:** Model Parameters for the Changes of EIVs Between Habitats

EIV	*p* value	DF versus SC	GR versus SC	*R*^2^
Soil reaction	0.861	0.590	0.798	0.01
Temperature	0.001	< 0.001	0.01	0.46
Moisture	0.004	0.058	0.001	0.40
Aeration	< 0.001	0.002	< 0.001	0.63
Light	< 0.001	< 0.001	< 0.001	0.84
Continentality	0.002	0.840	0.001	0.44
Humus content	< 0.001	< 0.001	< 0.001	0.80
Nutrients	0.408	0.295	0.239	0.08

DF = Deciduous Forests, GR = Grasslands and SC = Spruce Monoculture Plantations.

#### Functional Evenness

**Figure 5:**
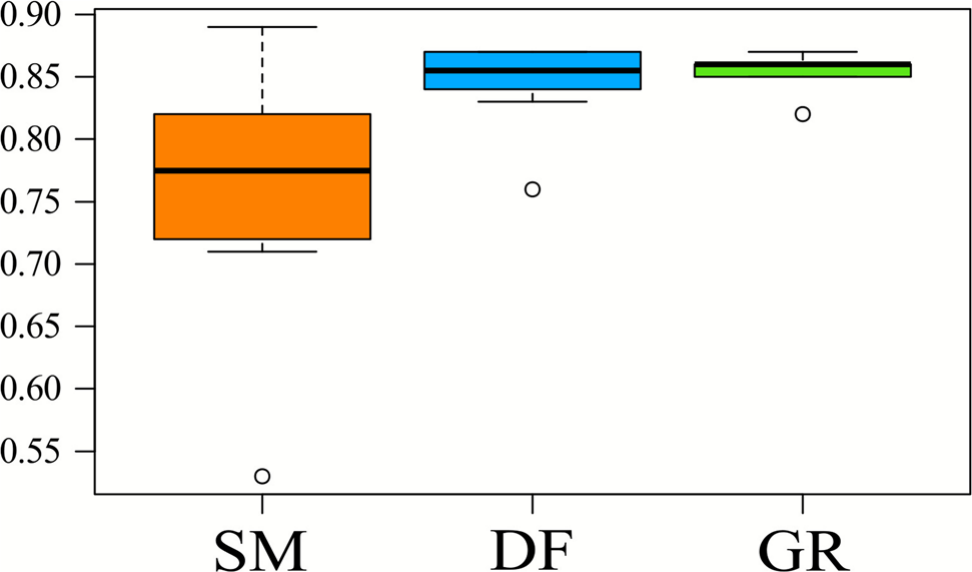
Box plots representing the levels of the functional evenness in EIVs among the three habitats (”SM”: spruce monoculture plantations, “DF”: deciduous forests, “GR”: grasslands). The values of functional evenness (on the y-axis) range from 0 to 1.

Results of the functional evenness across EIVs indicate differences in the overall distribution of ecological conditions among habitat types (*p *value = 0*.*014; Figure 5). In particular, functional evenness in spruce monoculture plantations was lower than that in deciduous forests (*p *value = 0*.*009) and grasslands (*p * value = 0*.*02). Deciduous forests and grasslands are also significantly different, despite having small differences (*p *value = 0*.*019 and *estimate* = 0*.*09).

### Soil Conditions

**Figure 6:**
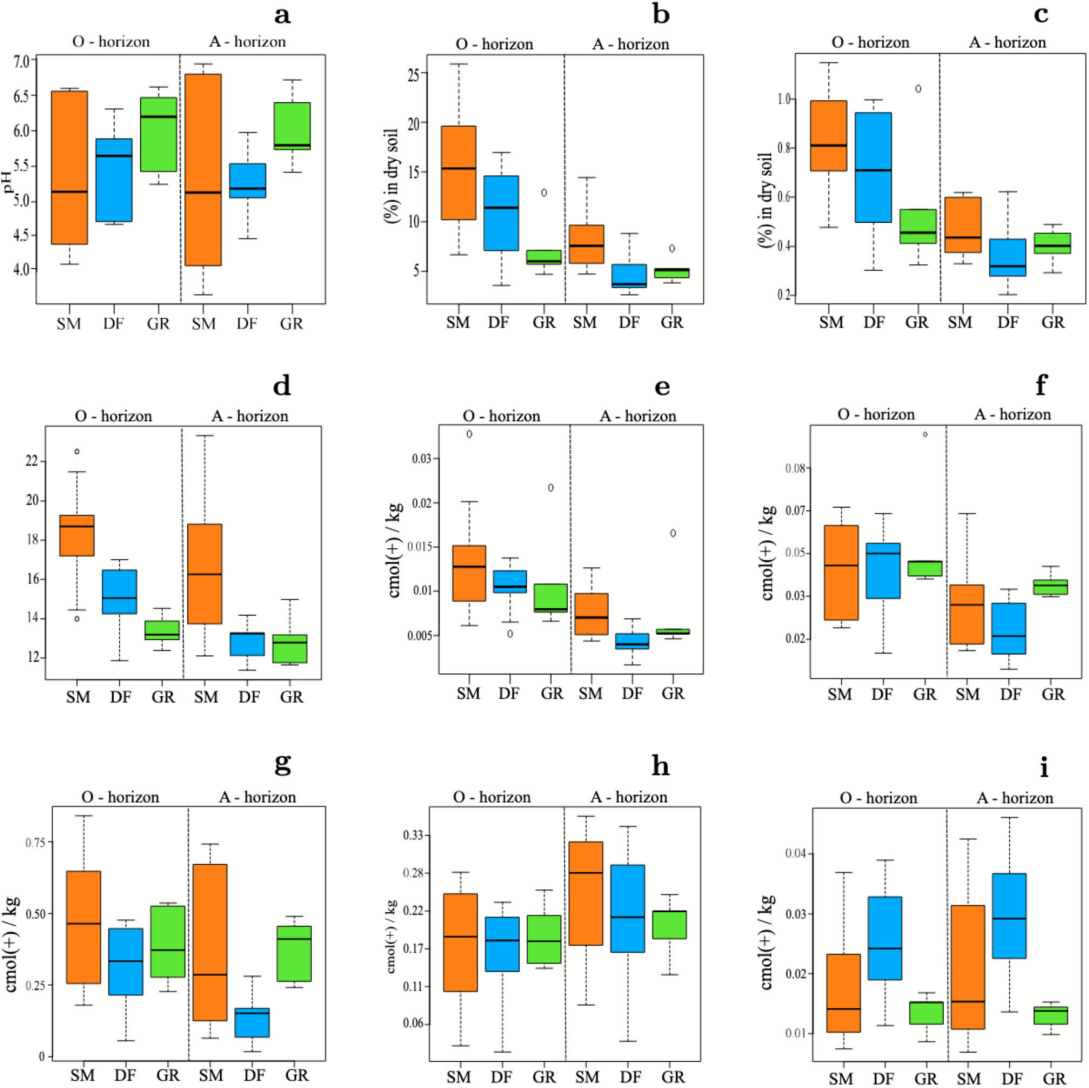
Box plots representing the differences of soil parameters for the three habitats, divided by horizon. On the x-axis are indicated the habitats (SM = spruce monoculture, DF = deciduous forest and GR = grassland). On the top of graphic are indicated the soil horizons (“O” horizon = litter, “A” horizon = organic mineral complexes). The y-axis shows the scales of each parameters taken into account.

Soil properties changed among habitats (Figure 6). In particular, pH (Figure 6a) was different among habitats overall for both horizons (*p *value = 0*.*048). Although the bedrock is an alkaline limestone, soil pH levels consistently lower than 7 indicate the absence of carbonates in the soil. For both horizons, the soil was more acidic in spruce monoculture plantations than in deciduous forests and grasslands. The soil organic carbon in the “O” horizon (Figure 6b) has shown differences between habitat types (*p* value < 0*.*001) with higher values in spruce monoculture compared to deciduous forest (*p *value = 0*.*02, *estimate* = 4*.*53) and grasslands (*p value* < 0*.*001, *estimate* = 8*.*4). Soil nitrogen (Figure 6c) does not change among habitats but only between horizons (*p* value < 0*.*001), being much higher in the “O” horizon than in “A” horizon. We also combined carbon and nitrogen in C/N (Figure 6d) given their ecological meaning as indicators of nutrient cycling rates in the soil (Gundersen and others 1998; Ollinger and others 2002) and found that habitat type (*p* value < 0*.*001) influenced soil C/N ratio in ”O” horizon. In particular, it was higher in spruce monoculture plantations than deciduous forests (*p *value = 0*.*006, *estimate* = 3*.*27) and grasslands (*p* value < 0*.*001, *estimate* = 5*.*82).

We also observed that soil exchangeable elements changed among spruce monoculture plantations and semi-natural habitats. In particular, potassium (Figure 6e) values changed among habitats (*p *value = 0*.*01) being higher in spruce monoculture plantations compared to deciduous forests (*p *value = 0*.*04, *estimate* =  − 1*.*39). Magnesium (Figure 6f) changed among habitats (*p *value < 0*.*001) as it was lower in spruce monoculture plantations than in grasslands (*p *value = 0*.*03, *estimate* = 0*.*97). Calcium (Figure 6g) changed among habitats overall (*p *value < 0*.*001), but there were no significant pairwise differences between habitat pairs. Aluminum (Figure 6h) and Iron (Figure 6i) were similar among habitats.

### Path Analysis

**Figure 7:**
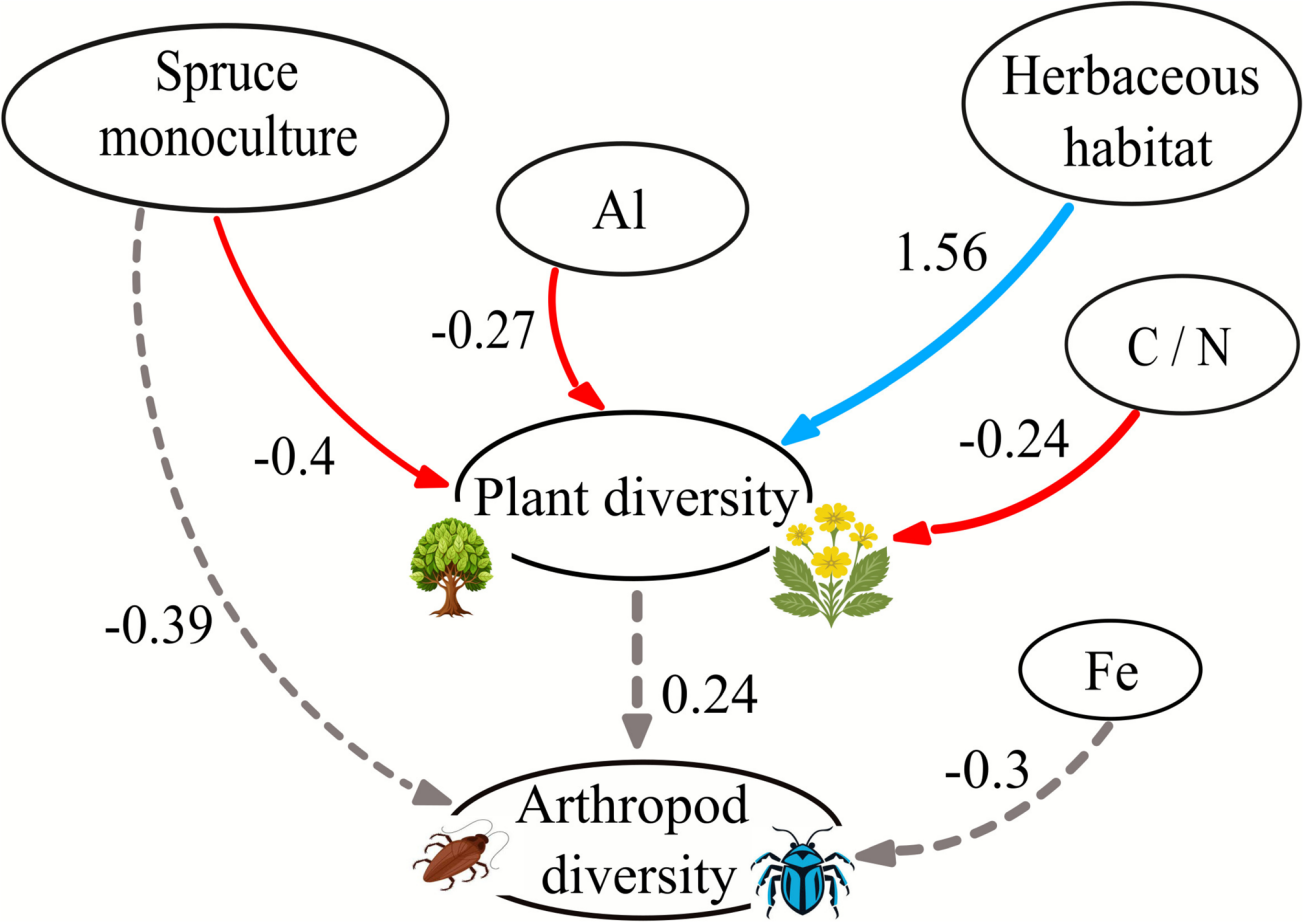
Path diagram resulting from the path analysis. The arrows represent the direct effects of a variable, labeled with the estimated coefficient. The nodes contain the variables.

The final model (Figure 7), resulting from the stepwise selection process with backward elimination, had a robust goodness of fit (Table 3).

**Table 3:** Comparison of Obtained Values with Acceptable Cut-off Criteria for Goodness-of-Fit Indices

Parameter	p(χ^2^)	GFI	AGFI	NFI	NNFI	CFI	RMSEA	SRMR	RFI
Treshold	> 0.05	≥ 0.90	≥ 0.90	≥ 0.90	≥ 0.95	≥ 0.95	< 0.08	≤ 0.08	≈ 1
Value	0.307	0.996	0.974	0.933	0.963	0.987	0.090	0.040	0.817

The first row indicates the threshold.

Plant diversity was influenced by both soil parameters and habitat properties. The presence of spruce monoculture has a negative impact on plant diversity, while grass-lands host the highest diversity. Higher levels of Al and C/N have negative effects on plant diversity. Arthropod diversity does not appear to be influenced by the variables selected by the model. Plant diversity does not have a significant effect on arthropod diversity, consistent with previous results.

## Discussion

We found that spruce plantations alter plant community composition, reduce plant diversity and functional evenness, and modify microhabitat conditions, particularly by reducing light availability and increasing litter deposition.

### Plant and Arthropod Diversity

Our comparative approach clarified how plant and arthropod communities respond to spruce monoculture plantations in the long-term, over *c* 100 years. As expected, grass-lands hosted the highest plant diversity. Less predictable, however, was the observed difference between the two forest types. This result may be explained by the absence of early-flowering species and the lack of other tree species in spruce monoculture plantations. Plants like geophytes are typical of deciduous forests, where they bloom before the canopy closure in spring. The evergreen nature of spruce prevents such phenological adaptation, likely reducing species richness (H´erault and others 2005; Manuzi and others 2025). Furthermore, the lack of seedlings and saplings of native tree species might be due to allelopathic effects associated with soil acidification and litter deposition (Aarrestad and others 2014).

While plant diversity was clearly still influenced by spruce plantations nearly 100 years after planting, arthropod diversity showed higher variation within habitats than between habitats. This result may be due to multiple factors. First, arthropods are mobile organisms that, in the absence of dispersal barriers, could easily move among the examined adjacent habitats (Anderson and others 2024). Furthermore, the minimum distance of 50 m between plots of different habitats might not have been enough to avoid the influence of adjacent habitat types. Second, arthropod diversity is not exclusively structured by habitat but also by trophic relationships, including plant diversity and predators (Losapio and others 2016; Ebeling and others 2018). A more diverse plant community typically supports a wider range of arthropods by providing more niches and food sources (Scherber and others 2010). However, arthropods are highly adaptable and can exploit a broad range of trophic resources (Eisenhauer and others 2019). Third, pitfall traps are effective for carabid beetles, arachnids, and soil-dwelling arthropods, such as springtails, aphids, and myriapods. The diversity of these organisms might be more closely linked to the biomass availability of prey and landscape structure than to plant diversity and soil ecological conditions. This sampling method might have excluded important groups of arthropods, such as herbivores and pollinators, as well as deadwood-dependent species that are more specialized than the examined generalist groups. Fourth, it is possible that the examined populations of arthropods are fairly resilient due to having recovered since the plantations were established nearly 100 years ago.

### Filtering Community Composition

The PLSDA confirmed distinct plant community compositions across habitats, validating the presence of three ecologically differentiated systems. Grasslands form a distinct group, consistent with their unique structure and the dominance of herbaceous species that are shade intolerant, as also reflected by the results of the light EIV analysis. The closer proximity of the deciduous forest and spruce monoculture ellipses reflects their shared structural features and overlapping species pools.

Further analysis revealed that the species diagnostic of spruce monoculture are nested within a broader group typical of deciduous forests, suggesting that spruce monoculture plantations represent a taxonomically impoverished subset of the native forest community (Manuzi and others 2025). These results provide evidence in support of our hypothesis that plantations of extra-range species reduce local plant biodiversity by filtering and impoverishing the species pool rather than by creating a distinct habitat that promotes the replacement of a few unique species. Importantly, species distinguishing native deciduous forests from spruce monoculture plantations, such as *Oxalis acetosella* and *Corylus avellana*, are not exclusive to temperate habitats; they also occur in boreal coniferous forests (Pignatti and others 2017). Therefore, the absence of distinct diagnostic species in spruce monoculture plantations underscores their lower ecological integrity and biodiversity value.

### Microhabitat and Ecological Indicator Values

Landolt EIVs provide further insights into microhabitat characteristics and highlight potential relevant ecological processes and limiting factors.

The soil reaction EIV showed that soil pH alone does not characterize plant communities. Yet, it is important to acknowledge that spruce monoculture plantations exhibit much greater variance, suggesting they support a diversified assemblage of species concerning the sole niche axis of soil pH tolerance. Higher values for soil aeration might reflect the specific rooting strategies associated with spruce trees, possibly increased by a warmer climate (Mauer and others 2009). The soil moisture EIV highlighted the influence of the forest canopy on the understory community.

Both deciduous forests and spruce plantations exhibit lower light availability than grasslands. Lower light implies lower incoming UV radiation, which causes a reduction in water loss due to evapotranspiration, ultimately decreasing the risk of heat stress and desiccation (Liu and others 2022). Furthermore, spruce evergreen canopy limits light penetration year-round (Stenberg and others 1999). This factor likely excludes early-flowering geophytes, which, instead, are prevalent in deciduous forests. The absence of these light-dependent species in spruce monoculture plantations reduces temporal niche availability and contributes to lower diversity (Manuzi and others 2025). Considering bioclimate, the continentality EIV was highest in grasslands, consistent with species typical of continental climates. Notably, the temperature EIV was lower in spruce monoculture plantations, possibly reflecting the cooler microclimate associated with coniferous canopy cover and plant species from higher-altitude ranges (Brna and others 2024).

Functional evenness differed significantly among habitats. Following Mason and others (2005) and Vill´eger and others (2008), functional evenness reflects the extent to which a community utilizes the available niche space. Lower values indicate that parts of the functional space are under-utilized, potentially reducing ecosystem productivity and resilience. Spruce monoculture’s low functional evenness suggests inefficiencies in resource use and greater vulnerability to disturbance. This may be due to spruce’s role in altering soil pH or to the exclusion of certain functional groups, such as early-flowering geophytes.

### Soil Conditions

Soil analysis confirmed that spruce monoculture alters soil structure, particularly in terms of soil organic carbon, *C*/*N* ratio, and potassium, indicating slower organic matter turnover. The higher retention of organic matter in forests compared to grass-lands probably arises from both the higher accumulation rates of organic matter due to litter deposition and the reduced rates of decomposition due to litter quality (Liao and others 2006). Accordingly, higher levels of the soil *C*/*N* ratio in plantations not only mirror the humus EIV but also indicate a slower turnover of organic matter in the spruce forest understory (Soldatova and others 2024).

Although we did not observe lower arthropod diversity in spruce monoculture, the higher SOC in the “O” horizon suggests a lower effect of bioturbation, probably associated with poor soil fauna activity (Guidi and others 2022). In addition, the slower organic matter turnover may be related to impoverished microbial communities, which were not assessed here, that might be less diverse in monoculture due to reduced plant inputs and lower microhabitat heterogeneity (Prasad and others 2021).

Soil pH in spruce monoculture showed high variability, ranging from 4.11 to 6.58. This is likely due to two ongoing phenomena. On the one hand, the acidic needle litter promotes podzolization and mineral leaching (Lundstr¨om and others 2000). On the other hand, the relatively high pH in some spruce monoculture patches may reflect the influence of base-rich minerals in the parent material or reduced leaching on steep terrain. (Bor˚uvka and others 2007). Both the exchangeable Al and Fe were not higher in the “O” horizon of spruce monoculture, as we expected as a consequence of soil acidification driven by spruce litter. However, the trend of Al suggests a potentially significant variation between spruce monoculture plantations and semi-natural habitats that might be achieved with a greater spatial coverage.

### Causal Relationships

The effects of exchangeable soil Al and soil C/N ratio on plant diversity highlight a potential ecophysiological mechanism underlying the impact of spruce plantations on plant communities via soil structure. The two parameters of soil organic carbon and soil nitrogen were higher in plantations compared to semi-natural habitats. High soil C/N ratio indicates a low turnover rate of organic matter, creating conditions of low nutrient availability and higher humic acidity. High Al content is also associated with acid soils that show strong chemical erosion of parent material (Lundstr¨om and others 2000) and might act as an ecological limitation for some plant species.

Although arthropod diversity was not influenced by any of the examined factors, it remains possible that key microhabitat properties were overlooked in this analysis. Furthermore, it is likely that other arthropod groups, such as pollinators and herbivores, will show different responses than those of soil fauna (Losapio and others 2016; Vujanovi´c and others 2023). Future studies should integrate multiple sampling methods to capture additional surface-active taxa, such as herbivores and pollinators, which are under-sampled by pitfall traps. Additional methods would be required to comprehensively assess canopy- or deadwood-dependent species (Wildermuth and others 2023; Ampoorter and others 2020). Future work could also refine arthropod assemblage analysis by addressing functional diversity and specific functional groups to provide additional insights into ecological responses to afforestation.

## Conclusions

Our study combines three complementary components at once: plants, arthropods, and soils, under a shared historical and environmental context, allowing us to assess the legacy effects of past management. We provide evidence for the long-lasting impacts of plantations on biodiversity and microhabitat conditions via the modification of key soil properties and ecological processes. As we hypothesized, Norway spruce monoculture has negative effects on plant diversity, confirming that the dominance of a single species outside his distribution range can be detrimental to biodiversity. Specifically, spruce monoculture plantations negatively impact plant communities and functional evenness through changes in soil organic matter turnover and humus accumulation. Although soil arthropod diversity appears less sensitive to habitat differences, possibly due to the high mobility of these animals, we cannot exclude the possibility that other arthropod groups, which are more strongly associated with plant diversity, such as pollinators or herbivores, might be equally impacted by plantations. Although spruce plantations favor plant species adapted to high humus content, low light, and low temperature, functional evenness results suggest that this habitat is less stable and efficient, which may compromise long-term ecosystem functioning. Taken together, plantation assemblages are essentially a degraded, impoverished subset of the deciduous forest community, contributing little to overall biodiversity.

Our study illustrates the ecological consequences of reforestation strategies that prioritize timber production over biodiversity and soil ecology. The patterns reported here reinforce the importance of historical legacies and plant–soil feedbacks in shaping present-day communities. Even native species, when planted outside their distribution range, can significantly affect local ecosystems. This insight has broader implications: if the extra-range native Norway spruce can degrade biodiversity, the risks posed by fast-growing, non-native species in global reforestation efforts may be even greater. Understanding the long-term ecological impacts of tree plantations can inform current and future reforestation and restoration programs to ensure that planting trees promotes biodiversity and ecosystem functions.

## Data Availability

The data and R code used to produce the results and figures reported in this manuscript are publicly accessible at: 10.5281/zenodo.18297702.
